# Enhancing Implant Position Accuracy in Guided Implantology: The Role of Drill Play Under Lateral Forces

**DOI:** 10.1002/cre2.70056

**Published:** 2024-12-15

**Authors:** Vasilios Alevizakos, Yannick Stryga, Constantin von See

**Affiliations:** ^1^ Research Centre for Digital Technologies in Dentistry and CAD/CAM, Department of Dentistry, Faculty of Medicine and Dentistry Danube Private University Krems an der Donau Austria

**Keywords:** drill play, guided implantology, implant positioning, lateral forces, surgical guide

## Abstract

**Objectives:**

This study investigates the impact of drill play on the precision of implant positioning under lateral forces in guided implantology.

**Materials and Methods:**

An in vitro experiment was conducted using artificial bone blocks and the SIC Invent‐guided surgery implant system. Custom drill guides were designed, and 3D‐printed, using three sleeve types: sleeveless, a big sleeve, and a small sleeve. Drillings were performed with varying lateral forces, and deviations in angle, depth, and position were measured.

**Results:**

Sleeveless guides showed the highest variability in deviations, with a maximum deviation of 3.92 mm under extreme lateral forces. Big sleeve guides provided the most consistent precision, with deviations ranging from 0.42 to 1.33 mm. Small sleeve guides showed moderate precision, with deviations from 0.14 to 2.17 mm. Higher lateral forces generally increased deviations across all guide types.

**Conclusions:**

Drill play significantly affects the precision of guided implant drilling, with lateral forces causing deviations from the planned implant position. Big sleeve guides offer better precision under lateral loads compared to sleeveless and small sleeve guides. Strict adherence to the drilling protocol is essential to minimize errors and ensure optimal implant positioning.

## 1 Introduction

The precise positioning of dental implants is crucial for both restorative and esthetic outcomes (Esquivel, Meda, and Blatz 2021). Advances in CAD/CAM (Computer‐Aided Design, Computer‐Aided Manufacturing) technologies have facilitated computer‐assisted treatment planning, allowing for the virtual planning of the desired implant position and optimal restoration before surgical intervention. Notably, developments in Digital Volume Tomography (DVT), associated software, and three‐dimensional (3D) additive manufacturing have significantly contributed toward achieving this precision (De Vos, Casselman, and Swennen 2009).

The technical support offered by these technologies provides numerous advantages to both patients and practitioners. Guided implant placement is less invasive and faster, resulting in greater patient comfort. The risk of operative complications, such as injury to critical mandibular structures, is reduced, and postoperative pain and swelling are minimized. Studies have demonstrated that implant survival rates are comparable between conventional and computer‐assisted implantation methods. However, guided surgical procedures show significantly lower marginal bone loss 5 years after implant loading compared to conventional techniques (Pozzi et al. 2014).

Digital treatment planning allows practitioners to establish a prosthetically optimized implant position preoperatively through a “Backward Planning” approach. The resulting positional data are transferred to the surgical procedure using a 3D‐printed guide. The precision outcomes of this process are generally favorable (Kalt and Gehrke 2008).

The accuracy in guided implant procedures is defined as the discrepancy between the virtual planned position and the actual position of the placed implant. The literature provides a cumulative enumeration of potential errors at various process stages (Block and Chandler 2009). Errors can arise during the digital workflow for creating custom surgical guides and during their application in the patient. The average deviation between planned and actual implant positions is about 1 mm, with accuracy often reported in terms of vertical or horizontal deviations or angulations (Tahmaseb et al. 2014).

During the use of a surgical guide, the drill ideally rotates parallel to the guide sleeve. To allow rotation, a gap between the drill and the guide sleeve is necessary, which can introduce another source of error (Van Assche et al. 2012). Lateral deviations occur when the drill is not precisely aligned within the guide sleeve. Without such a gap, unavoidable contact between the drill and the mechanical components of the guide could cause deformation.

Various design concepts for surgical guides and their mechanical components exist among manufacturers (D'Souza and Aras 2012). As guided implantation with surgical guides becomes increasingly common, understanding potential sources of error in planning and execution is essential. The current literature provides limited data on the effects of lateral forces on the accuracy and durability of mechanical components.

This study aims to investigate the impact of drill play on the precision of implant positioning.

## 2 Hypotheses

The null hypothesis posits that the precision of drilling will remain nearly constant despite increased lateral force due to the specific guidance provided by the implant system.

## 3 Materials and Methods

This study involves an in vitro experiment assessing the precision of guided implant drilling in artificial bone blocks under lateral force. The SIC Invent‐guided surgery implant system was utilized for the trials. The experimental setup simulated guided implant drilling with deviations in position measured under lateral loads.

### 3.1 Test Model Fabrication

For simulating drilling in bone‐like structures, artificial bone blocks from Sawbones (Washington, USA) were utilized. These bone blocks conform to ASTM standards, making them suitable for testing medical devices and materials. The blocks were obtained in various dimensions and densities and were subsequently cut to a uniform size (length = 7 cm, width = 4 cm, height = 2.5 cm) by Holzschnittfirma Sachseneder (Krems an der Donau, Austria). A bone block was scanned using the 3Shape D800 model scanner (Copenhagen, Denmark), resulting in a digital.stl (Standard Triangulation/Tesselation Language) file.

Using coDiagnostiX software (Dental Wings GmbH, Chemnitz, Germany), a customized drill guide template was designed. The software allows for 3D design of a drill guide incorporating the parameters of the SIC‐Guided Surgery Implant System.

**Figure 1:**
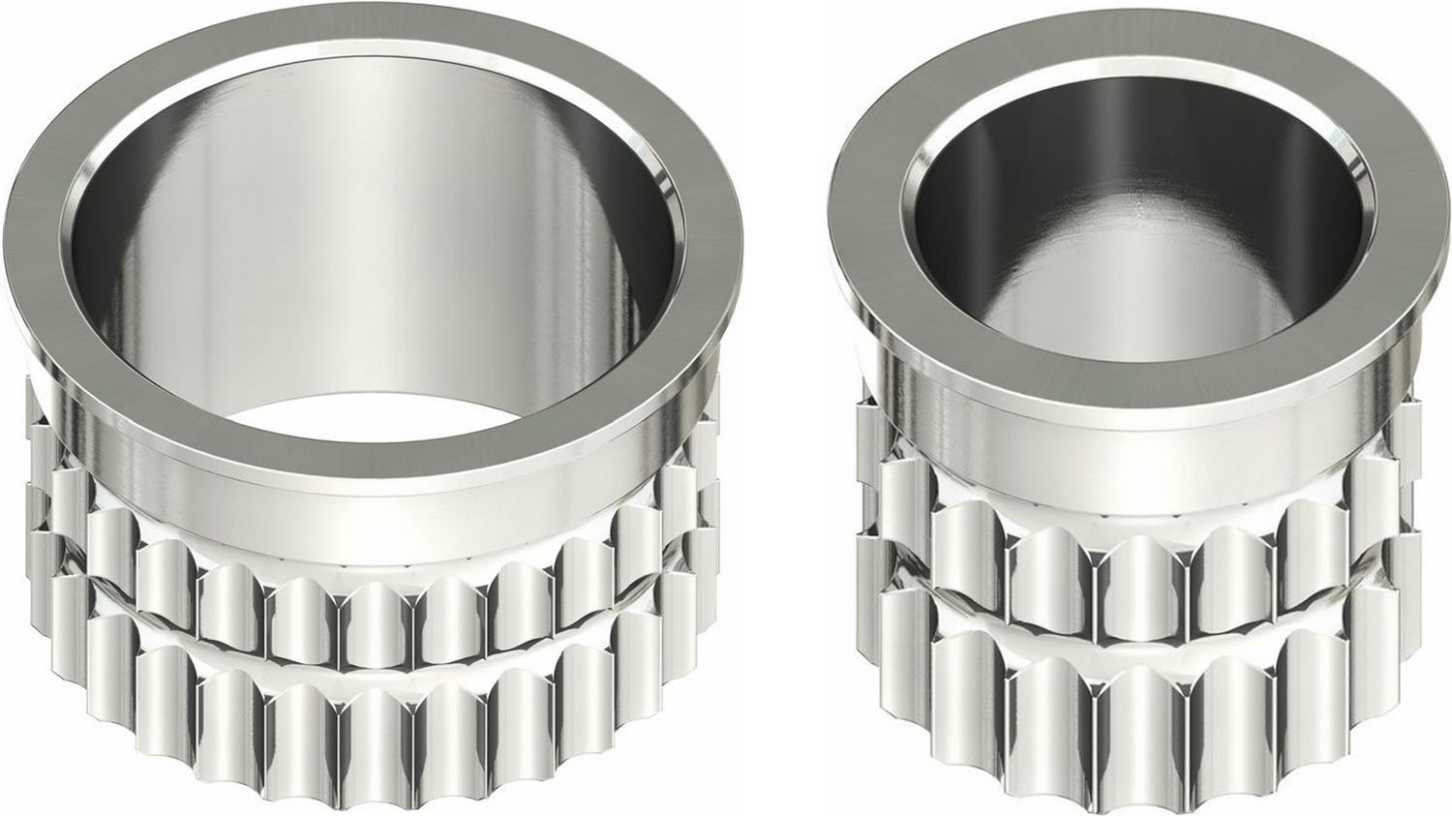
SIC‐Guided surgery sleeves (5.2 and 3.1 mm): The two core components of the SIC‐Guided surgery system are shown: the 5.2 mm sleeve (left) and the 3.1 mm sleeve (right).

**Figure 2:**
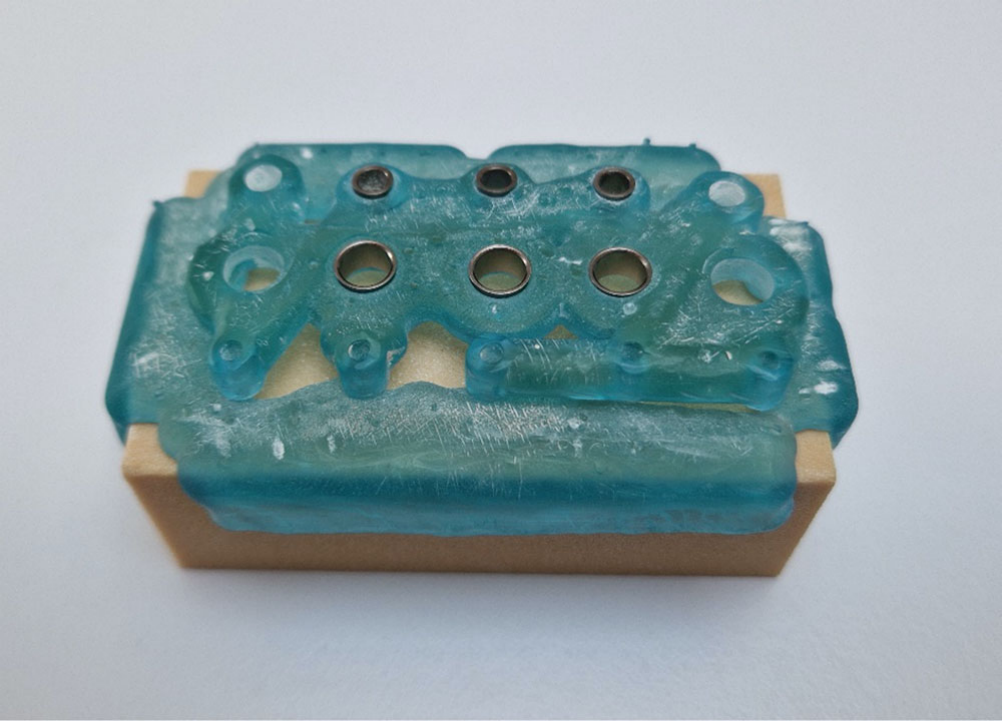
Custom‐fabricated guide positioned on the artificial bone block.

Three different sleeve types were chosen for the experiment. In the implant library of the software, the virtual sleeve for pilot drilling is referred to as SIC Pilot, with a diameter of 2.1 mm, suitable for drilling with the pilot drill without a metal sleeve. For drilling with metal sleeves, SIC sleeves of 3.1 and 5.2 mm diameters were used (Figure 1). The lengths of the sleeves were 6 mm (SIC Pilot), 5 mm (SIC 3.1), and 4 mm (SIC 5.2). The distance between the crest of the bone block and the drill sleeves was standardized at 3.5 mm. The virtual implant used was the SIC Invent AG SIC Ace with a length of 9.5 mm and a diameter of 4 mm. The implant shoulder was positioned 2.5 mm below the crest of the bone block, with its axis perpendicular to the surface. In total, five drillings were planned for each of the three different drill sleeves. The design of the drill guide was optimized for stability, featuring circular support on all four edges and directly on the surface to prevent unwanted rotational movement during drilling. The final design was processed using Bego CAMcreator Print (Bego GmbH, Bremen, Germany) for 3D printing with the Varseo S printer from Bego. The printing process took approximately 1 h, using VarseoWax Surgical Guide material from Bego. Following printing, metal sleeves were manually inserted into the fittings using a small amount of liquid VarseoWax. The printed drill guide was then post‐processed according to the manufacturer's instructions. Excess material was removed in an ultrasonic bath (Sonorex RK 100, Bandelin, Berlin, Germany) before post‐curing in the Otoflash curing unit (Bego) (Figure 2).

### 3.2 Experimental Setup

The experimental trial was conducted in a dental laboratory. Drilling was performed using a manually operated milling device (S2 Profi, Schick GmbH, Schemmerhofen, Germany) capable of vertical drilling at a fixed rotational speed.

A wooden beam was mounted on the platform of the milling device, and metal brackets sourced from a hardware store were securely fastened to its surface. Test models were attached to these brackets to allow rotational movement along their central axis.

To prepare the test models, the centers of the side surfaces were marked, and predrilled holes were created using a cordless drill (Bosch, Stuttgart, Germany). The models were then secured between the metal brackets using two screws. An additional screw was placed centrally on one side of each test model to serve as the application point for the lateral force. This setup allowed the bone block with the surgical guide to freely rotate around its axis during testing.

A fishing line was attached to the screw serving as the lateral force application point. At the opposite end of the fishing line, various weights were hung to apply lateral forces. A thin metal rod, placed between two additional metal brackets, acted as a guide and redirecting mechanism for the fishing line.

**Figure 3:**
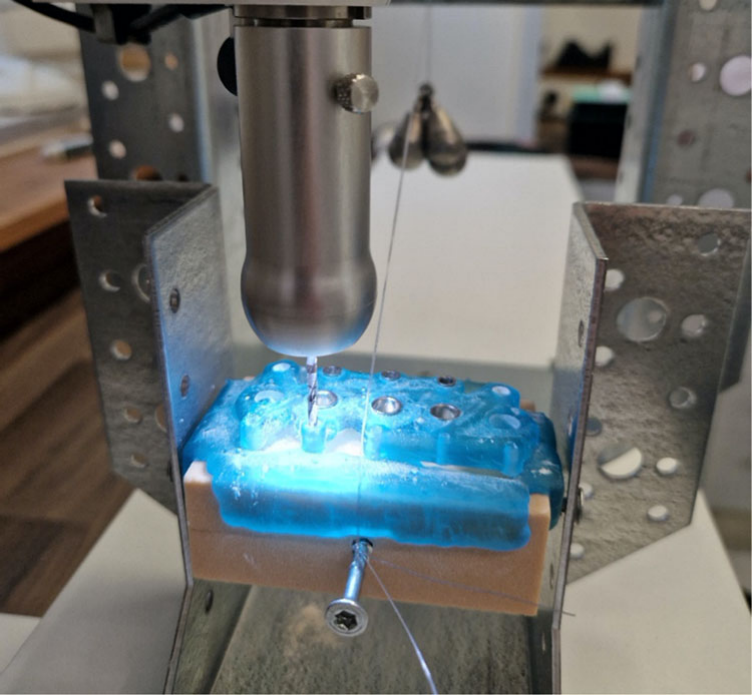
Experimental setup for guided implant drilling under lateral force application. The test model, secured between metal brackets, is mounted on a manually operated milling device. A fishing line is attached to the screw placed on the lateral side of the test model, simulating lateral forces through the hanging weights in the background. The surgical guide facilitates precise drill positioning, whereas the setup allows free rotation of the test model along its central axis.

This setup simulated a guided implantology procedure, acknowledging the realistic assumption that a drill may not always move perfectly vertically through the guide sleeve due to manual operation. This design aimed to replicate the potential tilting of the drill within the guide sleeve under the influence of lateral forces (Figure 3).

### 3.3 Procedure

Initial test drillings were performed without lateral force. With the milling machine precisely aligned vertically, drills were guided through the drill guide. A bone block was mounted on the platform, and the rotational speed was set to 800 revolutions per minute (rpm), as recommended by the SIC‐Guided Surgery technical manual. The drillings were conducted without water cooling. The vertical movement of the drills was smooth due to the resistance provided by the milling machine's hand wheel. Depth markings on the SIC‐Guided Surgery drills ensured a consistent drilling depth of 9.5 mm.

**Figure 4:**
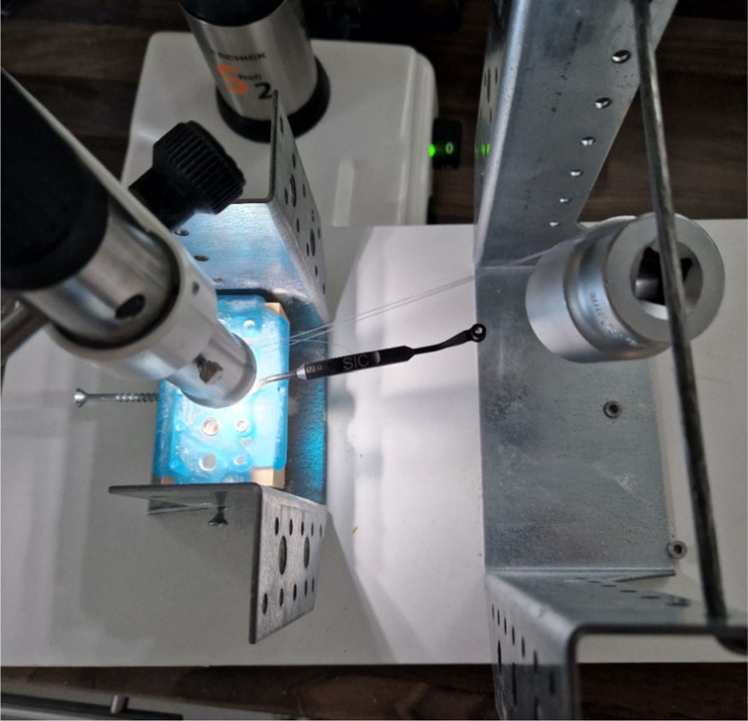
Top view of the experimental setup for guided implant drilling. The test model is secured between metal brackets, with a fishing line attached to a lateral screw to apply lateral forces via weights. The setup includes a surgical guide to ensure drill alignment and replicates deviations caused by lateral forces during manual implant procedures.

Subsequent drillings involved applying lateral force. For this phase, bone blocks were subjected to incremental weights: Blocks 1 and 2 were loaded with 100 g each, Blocks 3 and 4 were loaded with 200 g each, Blocks 5 and 6 were loaded with 300 g each, Blocks 7 and 8 were loaded with 400l̥g each, and Blocks 9 and 10 were loaded with 500 g each. A weight was attached to the fishing line, causing the bone block to rotate around its axis (Figure 4). The rotational speed of the drill was adjusted as needed to accommodate the increased resistance due to the misalignment in the drill sleeve. The pilot drill (2.0 mm diameter) and the expansion drill (3.75 mm diameter with yellow marking) were used as per the experimental setup. A minimum of three drillings per configuration was performed on each bone block, with each weight stage tested on an additional bone block. This resulted in a total of 88 drillings with lateral force, plus at least 11 additional drillings without lateral force or with extreme lateral force. Weights were incrementally increased by 100 g, with precise measurements taken using a scale. The maximum weight for the series was set at 500 g.

The analysis of the results was performed using Microsoft Excel (USA). For each test block, a table was created to record the values for drill depth and the deviation angle from the vertical axis. To calculate the deviation at the drill tip within the test block, a trigonometric function was applied. As the drill depth remains consistent regardless of deviation, the calculations utilized the trigonometric functions of an isosceles triangle. The deviation at the drill tip was determined using the following formula:

Deviation=2×DrillDepth×sin(Angle/2).

## 4 Results

**Figure 5:**
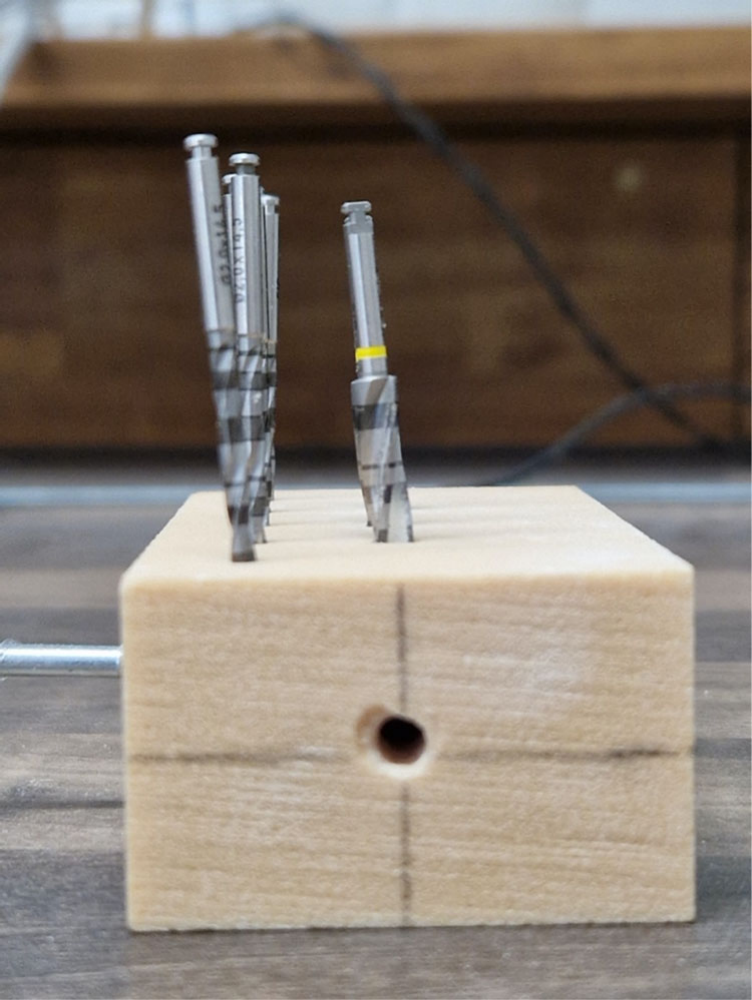
Angulation deviation after lateral load testing.

In this in vitro study, the precision of guided implant drilling in artificial bone blocks was evaluated under lateral forces, comparing three guide types: sleeveless (SL), a big sleeve (BS), and a small sleeve (SS). Drilling precision was assessed by analyzing the angle, depth, and deviation of the drills (Figure 5).

The results revealed that the type of guide significantly influenced precision. SL guides demonstrated the highest variability across all measured parameters, indicating reduced control and susceptibility to the effects of lateral forces, leading to inconsistent performance. In contrast, BS guides consistently achieved the highest precision, maintaining reliable performance even under moderate lateral forces. This made BS guides the most precise and dependable option. SS guides showed moderate precision. Although SS guides performed better than SL guides, they showed greater variability than BS guides, particularly, under stronger lateral forces.

When analyzing the angles, BS guides displayed the most stable performance, reflecting better control during drilling. SS guides performed moderately well but were more prone to variability, whereas SL guides showed significant outliers, indicating less precise orientation during drilling. Depth control followed a similar trend, with BS guides closely adhering to the target depths, SS guides showing moderate performance, and SL guides frequently over‐ or underdrilling, particularly, under lateral forces.

Overall, BS guides outperformed both SL and SS guides in terms of accuracy, stability, and reliability. The results highlight the critical role of guide type in achieving precise implant positioning under lateral forces. BS guides proved to be the most effective in minimizing deviations, whereas SL guides demonstrated the greatest variability, making them less suitable for precision‐sensitive procedures. SS guides offered a reasonable balance between performance and variability, serving as a middle‐ground option.

These findings underscore the importance of selecting the appropriate guide type to optimize implant positioning. BS guides provide superior control and precision, making them ideal for procedures requiring high accuracy. SS guides can serve as a viable alternative in less demanding scenarios, whereas SL guides should be used with caution due to their high variability. The summarized data in Table 1 provide further insight into these performance trends.

**Table 1:** Summary of the mean values and standard deviations for angle, depth, and deviation across different blocks and guide types. The table compares the precision of sleeveless (SL), big sleeve (BS), and small sleeve (SS) guides in terms of the drilling angle (°), depth (mm), and deviation (mm).

Block	Guide type	Mean angle (°) ± SD	Mean depth (mm) ± SD	Mean deviation (mm) ± SD
1	SL	0.86 ± 3.02	9.7 ± 1.3	0.13 ± 0.62
	BS	2.73 ± 0.35	10.3 ± 0.3	0.49 ± 0.07
	SS	3.33 ± 2.06	9.0 ± 0.3	0.51 ± 0.26
2	SL	−0.12 ± 3.82	11.0 ± 0.5	0.00 ± 0.75
	BS	4.60 ± 1.20	8.2 ± 0.3	0.70 ± 0.20
	SS	2.63 ± 4.08	8.7 ± 0.3	0.38 ± 0.65
3	SL	3.78 ± 1.85	10.2 ± 1.0	0.68 ± 0.35
	BS	4.30 ± 3.42	9.2 ± 1.3	0.62 ± 0.45
	SS	3.70 ± 1.92	9.0 ± 0.0	0.58 ± 0.30
4	SL	7.42 ± 3.48	9.0 ± 1.0	1.16 ± 0.44
	BS	4.43 ± 2.70	9.7 ± 0.3	0.75 ± 0.40
	SS	5.33 ± 1.54	7.0 ± 1.5	0.61 ± 0.16
5	SL	3.20 ± 1.47	10.0 ± 0.4	0.56 ± 0.26
	BS	4.80 ± 1.90	9.7 ± 0.3	0.82 ± 0.34
	SS	2.67 ± 1.15	8.0 ± 0.0	0.37 ± 0.16
6	SL	4.40 ± 2.00	9.5 ± 0.5	0.71 ± 0.33
	BS	7.83 ± 1.35	7.7 ± 1.5	1.04 ± 0.24
	SS	9.70 ± 7.80	7.2 ± 0.3	1.19 ± 0.90
7	SL	2.54 ± 1.53	11.8 ± 1.2	0.52 ± 0.30
	BS	7.83 ± 1.86	8.0 ± 0.8	1.08 ± 0.21
	SS	5.30 ± 2.92	10.5 ± 0.8	1.02 ± 0.51
8	SL	4.06 ± 0.80	11.8 ± 1.6	0.84 ± 0.22
	BS	3.83 ± 2.11	10.0 ± 1.2	0.67 ± 0.27
	SS	7.20 ± 3.58	9.8 ± 0.6	1.19 ± 0.46
9	SL	12.12 ± 4.54	11.5 ± 3.4	2.23 ± 1.39
	BS	6.70 ± 1.34	9.0 ± 0.5	1.00 ± 0.31
	SS	8.45 ± 3.36	8.3 ± 0.5	0.77 ± 0.11
10	SL	11.68 ± 3.40	9.6 ± 1.8	1.67 ± 1.43
	BS	7.57 ± 6.68	10.3 ± 1.1	1.93 ± 0.83
	SS	2.26 ± 0.99	9.2 ± 0.3	0.17 ± 0.30

## 5 Discussion

The results from our laboratory experiment provide insights into potential accuracy deviations associated with the use of drilling templates and guided surgery implant systems. Although numerous studies have previously investigated error sources and deviations in the manufacturing and application of drilling templates, this experiment focuses on the mechanical properties of both the drilling templates and the implant systems. Cassetta et al. estimate that 62.7% of deviations are attributed to the characteristics of guided implant systems and their associated sleeves and drills (Cassetta et al. 2012). During implantation, it can be challenging for the operator to move the drill exactly parallel to the axis of the drill sleeve. This highlights the importance of the mechanical components of the drilling template, as the combination of drill sleeve, handpiece, and drill can influence the accuracy of implant placement. Apostolakis and Kourakis demonstrate that a longer implant, combined with a high offset (distance between the bone surface and the drill sleeve) and a low height of the drill sleeve, results in greater deviations (Apostolakis and Kourakis 2018). Additionally, their study shows that increased drill play also contributes to larger deviations. Other studies have reported average deviations ranging from 1.2 to 1.6 mm, with Tahmaseb et al. reporting a maximum deviation of up to 7.1 mm (Tahmaseb et al. 2018). These studies vary in the design and the components used. In our laboratory experiment, we examined the impact of horizontal force on accuracy by testing three different drilling template scenarios: one without a metallic sleeve, with a length of 6 mm; a large metallic sleeve, with a length of 4 mm plus 1 mm guidance from the handpiece; and a small metallic sleeve, with a length of 5 mm plus 1 mm guidance from the handpiece. The highest deviations were observed in the scenario without a metallic sleeve, particularly at forces of 400 and 500 g, potentially due to increased material wear. The maximum deviation in this scenario was 3.92 mm, which is unacceptable for guided implantation. Deviations at forces under 400 g were generally up to 2.17 mm, which is consistent with literature values. Drillings through metallic sleeves showed similar results to those without a sleeve. Schneider et al. reported better precision with drilling protocols that did not use a metallic sleeve, but this experiment did not confirm these findings. Unusual values, such as negative deviations in Test Block No. 2 or minor errors with high horizontal forces in Test Blocks No. 9 and No. 10, were also observed. The causes of these outliers could not be investigated in this study, but technical errors during drilling are suspected. Extreme horizontal loads of 1 and 2 kg caused significant damage to the drilling template, handpiece, and drill sleeve. These high loads led to noticeable heat generation, wear, and loud friction noises.

The technical execution of the experiment followed strict guidelines for the materials and machinery used, with essential steps overseen by experienced technical specialists and clinicians. The drill speed played a crucial role in the experiment. Increasing lateral force required an increase in drill speed. Although drilling through the sleeves at nearly parallel angles allowed maintenance of the recommended speed of 800 rpm, significant increases in speed were needed to continue drilling, leading to substantial friction effects, heat development, and material abrasion. The wear behavior of the drilling template was not investigated in detail but likely influenced the results, especially under higher lateral forces. Given that the manufacture of individual drilling templates might also involve errors, assessing the quality improvement of the results is challenging. The design of the drilling template was primarily optimized for the experimental setup, with criteria such as layer thickness and stability significantly influencing its form. In practice, the design of the drilling template is affected by the anatomical structures in the mouth and may differ from the template used in this experiment. Different bone blocks of varying densities were used randomly during the experiment. The impact of block density on the results was not investigated. No obvious deviations related to block density were observed.

Technical instructions were followed strictly, with oversight from experienced specialists. The drill speed played a crucial role; increased lateral force required higher speeds, leading to friction and wear on the components. The study did not specifically investigate the wear and tear of the guide, but acknowledged its impact, especially under high lateral forces. The design and material of the guide influenced its performance, and variations in bone block density did not significantly affect the results.

However, this study provides valuable insights into the impact of drill play and lateral forces on guided implantology precision, but the limitations must be acknowledged.

First, the controlled laboratory conditions do not fully replicate clinical scenarios, where variables such as patient movement, saliva, blood, and bone density variability could significantly influence outcomes. Additionally, the artificial bone blocks used, although standardized and consistent, do not fully mimic the structural and anatomical complexity of human bone.

The lateral forces in this study were simulated using incremental weights, which may not accurately represent the dynamic and variable forces encountered during manual drilling in clinical practice. Moreover, the findings are specific to the SIC Invent‐guided surgery system and its custom drill guides, limiting the generalizability to other implant systems with different designs or tolerances.

Material wear and heat generation, particularly, under higher lateral forces, were not systematically analyzed. Both factors could affect the durability of the guides and the precision of drilling over time. Similarly, the study excluded human factors such as operator skill, technique, and fatigue, which are critical in real‐world outcomes.

Finally, the study focused exclusively on mechanical precision and did not assess clinical outcomes such as implant stability, osseointegration, or patient satisfaction. The sample size, though sufficient for preliminary findings, limits the statistical power and robustness of the conclusions.

Despite these limitations, the study provides a strong foundation for understanding mechanical factors influencing guided implantology precision. Future research should aim to address these gaps by incorporating clinical variables, evaluating material durability, and exploring the long‐term implications of drilling deviations.

## 6 Conclusion

This study has shown that drill play within a surgical guide significantly affects drilling precision. Lateral forces during drilling cause deviations from the planned implant position. Using the guided implant system from SIC Invent GmbH, precision deviations similar to those reported in the literature were observed under low lateral loads. Strict adherence to the drilling protocol helps prevent major errors even under higher loads.

Understanding the impact of technical parameters on precision is essential when designing surgical guides. Users should be aware of potential errors related to parameters like drill sleeve length and type, drill length, and distance to the bone. These insights aid in planning safe distances to critical anatomical structures.

Although this study offers valuable insights into the application quality of the implant system, further research is needed to explore questions about drill sleeves and efficient workflows. Drill play will remain a potential source of error in future studies on guided implantation precision. The user's experience with the system is crucial in minimizing these errors.

For optimal results in guided implantology, it is important to closely follow technical instructions during both guide construction and implantation.

## Author Contributions

Vasilios Alevizakos contributed to the conceptualization and design of the study, conducted the experiments, and played a major role in drafting the manuscript. Yannick Stryga was responsible for the development of the methodology, data analysis, and also contributed significantly to the validation and review of the manuscript. Constantin von See provided supervision and project administration, secured funding, and was instrumental in the final review and editing of the manuscript. All authors read and approved the final version of the manuscript.

## Ethics Statement

We declare to maintaining the highest standards of ethics and integrity in all aspects of our work.

## Consent

The authors have nothing to report.

## Conflicts of Interest

The authors declare no conflicts of interest.

## Data Availability

The data that support the findings of this study are available from the corresponding author upon reasonable request.
